# Dysregulated ribosome quality control in human diseases

**DOI:** 10.1111/febs.17217

**Published:** 2024-07-01

**Authors:** Tom McGirr, Okan Onar, Seyed Mehdi Jafarnejad

**Affiliations:** ^1^ Patrick G. Johnston Centre for Cancer Research Queen's University Belfast UK; ^2^ Department of Biology, Faculty of Science Ankara University Turkey

**Keywords:** proteostasis, ribosome collisions, ribosome quality control, ribosome stalling, RQC

## Abstract

Precise regulation of mRNA translation is of fundamental importance for maintaining homeostasis. Conversely, dysregulated general or transcript‐specific translation, as well as abnormal translation events, have been linked to a multitude of diseases. However, driven by the misconception that the transient nature of mRNAs renders their abnormalities inconsequential, the importance of mechanisms that monitor the quality and fidelity of the translation process has been largely overlooked. In recent years, there has been a dramatic shift in this paradigm, evidenced by several seminal discoveries on the role of a key mechanism in monitoring the quality of mRNA translation – namely, Ribosome Quality Control (RQC) – in the maintenance of homeostasis and the prevention of diseases. Here, we will review recent advances in the field and emphasize the biological significance of the RQC mechanism, particularly its implications in human diseases.

## Graphical abstract

Ensuring the synthesis of full‐length, correctly folded proteins is vital for maintaining homeostasis. If a translating ribosome stalls, the trailing ribosomes collide into it, which if left unresolved leads to deleterious effects on the cell due to the accumulation of protein aggregates. Ribosome Quality Control (RQC) detects the collided ribosomes and removes the aberrant mRNA and nascent peptide. Conversely, dysfunctional RQC or excessive ribosome collisions that overwhelm RQC can lead to proteotoxicity and diseases.

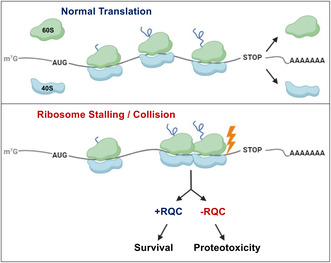

**Abbreviations**
40S: eukaryotic small ribosomal subunit
4E‐BP1: eIF4E‐binding protein 1
4EGI‐1: eIF4E/eIF4G Interaction inhibitor‐1
4EHP: eIF4E‐Homologous Protein
60S: eukaryotic large ribosomal subunit
80S: eukaryotic ribosome
8‐OxoG: 8‐OxoGuanine
A site: amino acid site
ABCE1: ATP binding cassette subfamily E member 1
AD: Alzheimer's disease
ADDS: aldehyde degradation deficiency syndrome
AKT: protein kinase B
ALDH2: aldehyde dehydrogenase 2 family member
ALS: amyotrophic lateral sclerosis
ANKZF1: ankyrin repeat and zinc finger peptidyl tRNA hydrolase 1
APP: amyloid precursor protein
ASCC: activating signal co‐integrator 1 complex
ASCC2: activating signal co‐integrator 1 complex subunit 2
ASCC3: activating signal co‐integrator 1 complex subunit 3
ATP: adenosine triphosphate
Aβ: β‐amyloid
BGLF2: Epstein–Barr virus tegument protein
C9ORF72: chromosome 9 open reading frame 72
CAT: C‐terminal addition to translation
CD8+: cluster of differentiation 8
CDK5RAP3: CDK5 regulatory subunit associated protein 3
cGAS: cyclic GMP‐AMP synthase
CSF2: colony‐stimulating factor 2
CTP: cytidine triphosphate
Cue2: coupling of ubiquitin conjugation to ER degradation
ddhCTP: 3′deoxy‐3′, 4′‐didehydro‐CTP
DKC1: dyskerin pseudouridine synthase 1
E site: exit site
EBNA1: EBV nuclear antigen 1
EBV: Epstein–Barr virus
EDF1: endothelial differentiation‐related factor 1
eEF: eukaryotic elongation factor
eIF: eukaryotic initiation factor
EMT: epithelial‐mesenchymal transition
ERAD: endoplasmic reticulum‐associated protein degradation
eRF: eukaryotic release factor
FAT10: HLA‐F adjacent transcript 10
FMRP: fragile X mental retardation protein
FXS: fragile X syndrome
GABAA: γ‐aminobutyric acid type A
GCN1: general control of amino‐acid synthesis 1
GIGYF2: GRB10 interacting GYF protein 2
GPX4: selenoprotein glutathione peroxidase 4
GTP: guanosine‐5′‐TriPhosphate
GTPBP2: GTP binding protein 2
HBSL1: Hsp70 subfamily B suppressor 1‐like
HD: Huntingtin disease
HRAS: HRas proto‐oncogene
HSP60: heat shock protein 60
HTT: Huntingtin
IFI44: interferon induced protein 44
IFIT2: interferon induced protein with tetratricopeptide repeats 2
Ifnb1: interferon beta 1
iPSCs: induced pluripotent stem cells
iRQC: initiation ribosome quality control
ISGs: interferon‐stimulated genes
ISR: integrated stress response
LDL‐C: low‐density lipoprotein‐cholesterol
LDL‐R: low‐density lipoprotein receptor
LGR5: leucine‐rich repeat‐containing G‐protein coupled receptor 5
LRIG1: leucine‐rich repeats and immunoglobulin‐like domains protein 1
LTN1: listerin E3 ubiquitin protein ligase 1
MHC: major histocompatibility complex
mHTT: mutant HTT
miRISC: microRNA‐induced silencing complex
MKRN1: Makorin Ring Finger Protein 1
mRNA: messenger ribonucleic acid
mtHSP70: mitochondrial heat shock protein 70
mTORC1: mammalian/mechanistic target of rapamycin complex 1
MTS: mitochondrial targeting sequence
N4BP2: NEDD4 binding protein 2
NEMF: nuclear export mediator factor
NGD: no‐go decay
NPLOC4: nuclear protein localization protein 4 homolog
NRF2: NFE2‐related factor 2
NSP1: non‐structural protein 1
NSP2: non‐structural protein 2
ORF: open reading frame
OTUD3: OTU deubiquitinase 3
P site: polypeptide site
PABPC1: poly(A)‐binding protein cytoplasmic 1
PAMP: pathogen‐associated molecular patterns
PCSK9: proprotein convertase subtilisin/kexin type 9
PD: Parkinson's disease
PELO: Pelota
PIC: pre‐initiation complex
PINK1: PTEN induced kinase 1
PRRs: pattern recognition receptors
PUS7: pseudouridine synthase 7
RACK1: receptor for activated C kinase 1
RIG‐I: retinoic acid‐inducible gene I
RING domain: RING‐type zinc finger domain
RNase: Ribonuclease
RNF10: ring finger protein 10
ROS: reactive oxygen species
RPL26: ribosomal protein L26
RPS10: small ribosomal subunit protein eS10
RQC: ribosome quality control
rRNA: ribosomal ribonucleic acid
RSAD2: radical S‐adenosyl methionine domain containing 2
RSR: ribotoxic stress response
SAM: sterile alpha motif
SAPK: stress‐activated protein kinase
SARS‐CoV‐2: severe acute respiratory syndrome coronavirus 2
SLFN11: Schlafen family member 11
STING: stimulator of interferon response cGAMP interactor
TCF25: transcription factor 25
TOM: translocase of the outer membrane
TOM1: target of Myb1
TRIP4: thyroid hormone receptor interactor 4
tRNA: transfer ribonucleic acid
TTC3: tetratricopeptide repeat domain 3
TTP: tristetraprolin
UBE2D3: ubiquitin conjugating enzyme E2 D3
UFDL1: ubiquitin fusion degradation protein 1 homolog
UFL1: UFM1 specific ligase
UFM1: ubiquitin‐fold modifier 1
USP21: ubiquitin specific peptidase 21
UTR: untranslated region
UV: ultraviolet
VCP: valosin‐containing protein
vDUB: viral deubiquitinating enzyme
Xrn1: 5′‐3′ exoribonuclease 1
ZAK: sterile alpha motif and leucine zipper containing kinase AZK
ZNF598: zinc finger protein 598

## Introduction

Eukaryotic mRNAs are commonly modified with a m7GpppN cap at their 5′ end, which protects the mRNA against degradation by 5′ to 3′ endonucleases and facilitates the initiation of mRNA translation. To initiate translation, the 43S pre‐initiation complex (PIC) composed of a 40S small ribosomal subunit, aminoacylated initiator methionine tRNA, GTP, and the eukaryotic initiation factor 2α (eIF2α) is recruited to the mRNA, along with several other initiation factors. The eIF4F complex, assembled from the scaffolding protein eIF4G, the RNA helicase eIF4A, and the cap‐binding protein eIF4E, is responsible for the recruitment of PIC to the cap via the eIF3 complex. eIF3 simultaneously binds to eIF2α, the 40S ribosome, and eIF4G. Subsequently, PIC scans the 5′ UnTranslated Region (5′ UTR) of the mRNA until it detects the Start codon. This is followed by the joining of the 60S ribosomal subunit, formation of the complete 80S ribosome, and beginning of the elongation phase of translation (reviewed in more detail in ref. [1]).

Elongation proceeds by addition of amino acids to the growing nascent polypeptide chain through the recruitment of the cognate aminoacyl‐tRNAs to the A site of the ribosome. The elongation factors 1 and 2 (eEF1 and eEF2) and eIF5A assume pivotal roles in this process. Initially, eEF1 orchestrates the binding of the aminoacyl‐tRNA to the A site on the ribosome. Subsequently, the polypeptide chain from the peptidyl‐tRNA in the P site is transferred to the aminoacyl‐tRNA in the A site while eIF5A contributes to substrate positioning for peptide bond formation [2, 3, 4]. Following this, the tRNA from the A site, now bound to the nascent polypeptide, translocates to the P site of the ribosome, while the newly deacylated tRNA in the P site translocates to the E site [5]. Once a new aminoacylated‐tRNA enters the A site, the uncharged tRNA exits the ribosomal E site and this elongation cycle continues until a stop codon is reached. During termination, the eukaryotic release factor 1 (eRF1) in the eRF1‐eRF3‐GTP termination complex recognizes and binds to the stop codon in the ribosomal A site [6]. Subsequently, the ATP‐binding cassette family ABCE1 facilitates the dissociation of the 60S from the 40S [7].

Dysregulated general or transcript‐specific translation has been linked with a multitude of diseases such as cancer [8] and neurodevelopmental disorders [9]. The past decade has seen a notable increase in our understanding of the mechanisms that regulate translation elongation stimulated by interest in addressing the potential negative consequences arising from impediments to ribosomal movement along the ORF. In this context, we will review recent findings that link ribosome quality control (RQC), a crucial mechanism of monitoring translation elongation, to human diseases.

### Ribosomal pausing and stalling

**Fig. 1:**
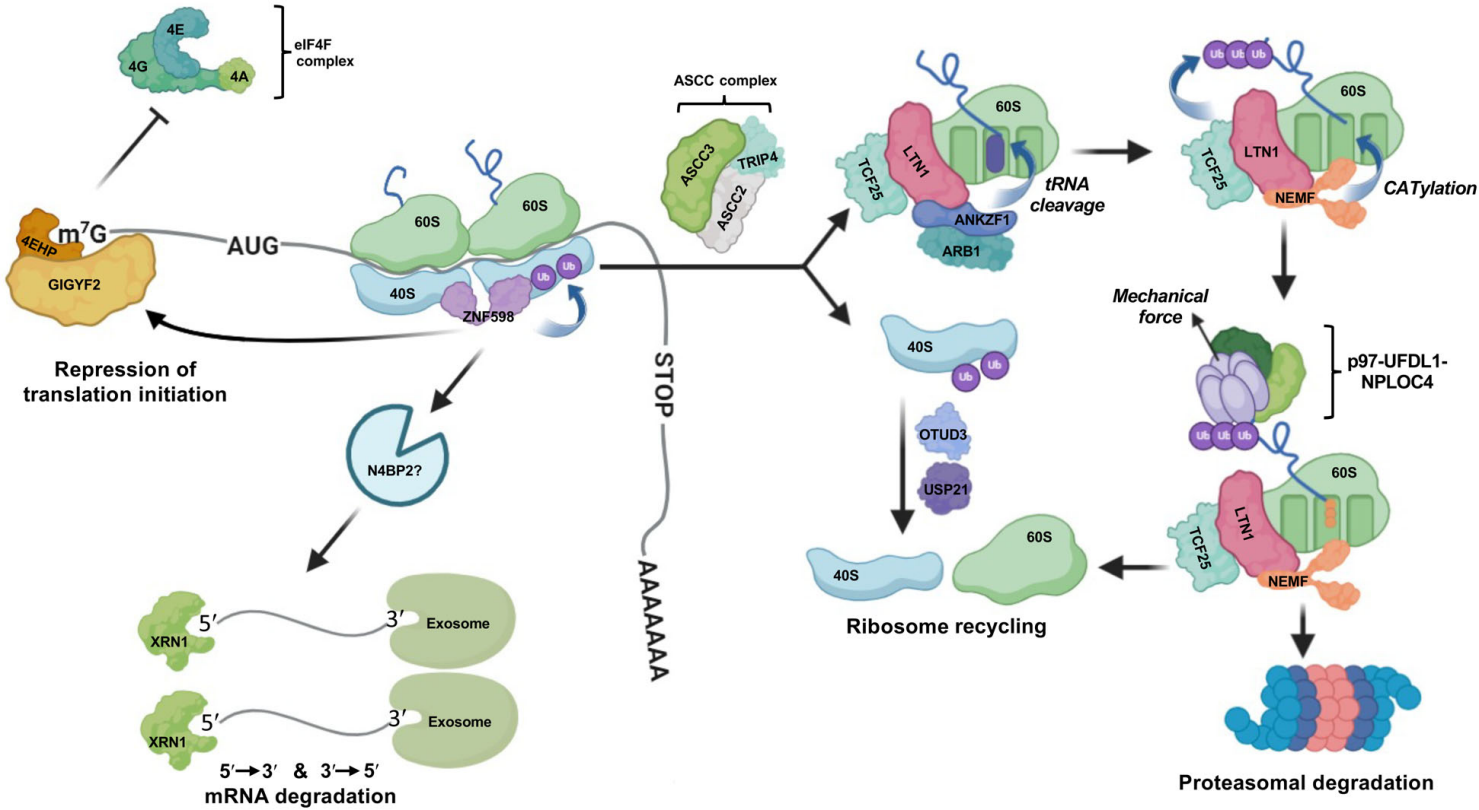
Ribosomal Quality Control (RQC) mechanism. When a translating ribosome stalls, for instance due to damaged nucleotides, the trailing ribosome collides into the stalled ribosome. These collision events are detected by the E3 ubiquitin ligase and RNA‐binding protein, ZNF598, which induces ubiquitination of several small ribosomal subunit (40S) proteins of the leading ribosome. Subsequently, the GIGYF2/4EHP translational repressor complex is recruited by ZNF598. 4EHP displaces the eIF4F complex on the 5′ cap, thereby preventing further rounds of translation initiation on the mRNA. The ASCC complex binds to the leading ribosome following its ubiquitination by ZNF598, splitting the ribosome into the ubiquitinated 40S and peptidyl‐tRNA‐bound 60S subunits through the helicase activity of ASCC3. Deubiquitination of the 40S subunit by OTUD3 and USP21 enables its recycling into the cellular pool. The RQC complex binds to the peptidyl‐tRNA‐bound 60S, and ANKZF1 is stimulated by ARB1 to cleave the CCA‐ bond between the nascent peptide and the tRNA, enabling their dissociation. NEMF displaces the ANKZF1 bound to 60S and promotes C‐terminal addition to the translation tail (CATylation) of the nascent peptide, while LTN1 polyubiquitinates the N‐terminal end of the nascent peptide. p97 is recruited to the ubiquitinated nascent peptide and, together with the NPLOC4 and UFDL1 cofactors, generates mechanical force through ATP hydrolysis that pulls the nascent peptide through the central pore of p97 and out of the 60S. The nascent peptide is subsequently shuttled to the proteasome and presented for degradation by p97, while the 60S subunit is recycled. The mRNA is cleaved by an endonuclease, Cue2 in yeast, and potentially its human homolog N4BP2, and subsequently degraded by the exonuclease activity of XRN1 (5′ to 3′) and the Exosome (3′ to 5′).

The rate of elongation is not constant and can vary due to numerous factors such as low codon optimality [10]. The sequence context in which codons appear in the mRNA, as well as structural features of the mRNA also influence elongation rates [11]. Suboptimal codons can reduce the elongation rate by transiently pausing the ribosome which can impact the efficient co‐translational folding of the nascent peptide in certain mRNAs [12, 13]. This transient ribosomal pausing may also be required for the efficient recruitment of the translating ribosome and nascent peptide to the endoplasmic reticulum [14]. However, elongation pauses are not always transient or part of normal physiological functions. For example, the presence of uninterrupted adenine repeats (poly(A) tracts) within the ORF leads to prolonged ribosome stalling [15]. If unresolved, this leads to a collision between the stalled ribosome and the trailing ribosomes. Ribosome stalling and collision could harm the cell by depleting the number of productive ribosomes and generating truncated or aberrant proteins through frameshifting [16, 17] as well as toxic protein aggregates [18]. Ribosome collisions occur commonly in normal conditions as evidenced by transcriptome‐wide analysis of ribosome collisions using disome‐profiling assay, which estimated that 11% of mRNAs have at least one ribosome collision site [19]. The evolutionarily conserved RQC mechanism (Fig. 1) resolves these by recycling the ribosomal subunits and removing the mRNA and nascent peptide.

### Detecting the collided ribosomes

The “canonical” RQC mechanism is initiated by ZNF598, an RNA‐binding and E3 ubiquitin ligase protein that detects the collided ribosomes and promotes the preferential ubiquitination of the eS10 (RPS10), eS3, and eS20 ribosomal subunits by the E2 ubiquitin ligase UBE2D3 [16, 20]. Structural studies of polysomes have shown that the trailing ribosome has an unrotated state, with a peptidyl tRNA in the P site, and the leading ribosome has a rotated state with a peptidyl tRNA in the P/A sites and an uncharged tRNA in the P/E sites [21]. This unique state of the ribosomal sites during translation may facilitate the recognition of ribosomal collisions by ZNF598. RACK1 contributes to the stabilization of the interface between the 40S subunits of the two collided ribosomes and facilitates the ubiquitination of ribosomal proteins by ZNF598 [22].

### Ribosome splitting and recycling

Following 40S ubiquitination by ZNF598, the ASCC complex consisting of ASCC3, ASCC2, and TRIP4, is specifically recruited to the leading 80S ribosome and splits it into the peptidyl‐tRNA 60S and ubiquitinated 40S subunits [23, 24, 25]. Although the precise mechanism of ribosomal splitting in mammals is not known, Slh1, the yeast ortholog of ASCC3, was shown to exert a pulling force on the mRNA through its helicase activity that promotes ribosomal dissociation [26]. Alternatively, another splitting complex composed of HBSL1 and PELO selectively binds to and splits stalled ribosomes with an empty A site at the mRNA 3′ end [27, 28]. HBSL1‐PELO complex binds ribosomes in a similar way to the canonical release factor eRF1 and eRF3 while also being dependent on ABCE1 activity for splitting [29]. However, it lacks the catalytic activity of eRF1 needed to hydrolyse the peptidyl‐tRNA bond and so produces a peptidyl‐tRNA‐bound 60S subunit [30]. The 60S subunit is therefore obstructed by the nascent peptide conjugated to tRNA and requires further processing before it can be recycled. Ubiquitination of the 40S subunits is reversed by the action of the deubiquitinase enzymes OTUD3 and USP21, which enables recycling of the 40S subunit [31].

### Degradation of the nascent peptide

Dissociation of the nascent peptide from the 60S unit and its subsequent degradation is facilitated by the orchestrated function of several subunits of the “RQC” complex. The N terminus of LTN1 specifically binds to the 60S ribosome at the inter‐subunit interface [32, 33]. This induces conformational changes in the LTN1 middle domain that positions the C‐terminal RING domain for K48‐linked ubiquitination of the nascent peptide and targets it for proteasomal degradation [32, 33, 34]. TCF25 promotes the K48‐linked polyubiquitination of the nascent peptide by LTN1 [35, 36]. NEMF affects the LTN1‐mediated polyubiquitination of the nascent peptide by pinning LTN1's N terminus against the 60S, thereby increasing the affinity of their interaction [32, 37]. Before the LTN1‐ubiquitinated nascent peptide can be efficiently degraded by the proteasome it must be removed from the 60S. This is achieved by addition of a poly‐Alanine C‐terminal extension to the stalled tRNA‐bound peptides through a non‐canonical elongation reaction called C‐terminal addition to translation (CAT) tailing. NEMF binds to and positions tRNA‐Ala within the A site, directing the ribosome to elongate the Alanine extension, which subsequently acts as a degron motif [38].

Freeing the 60S subunit from the nascent peptide begins with dissociation of the tRNA from the peptide. ANKZF1 binds to the A site, displacing NEMF and positioning its catalytic loop toward the 3′ CCA region of the tRNA which binds the nascent chain [39, 40]. ANKZF1 is then stimulated by its cofactor Arb1 to cleave the bond between the nascent chain and tRNA, thus producing a 3′ CCA nascent peptide and a cleaved tRNA [39, 41]. The nascent peptide is then removed from the 60S subunit by the p97‐UFDL1‐NPLOC1 complex, which binds to K48‐linked ubiquitin chain on the nascent peptide [35, 37, 42, 43]. P97 pulls the peptide chain through its central pore [42, 43, 44, 45], exposing a newly unfolded region at the other end of the pore to the proteasome [44, 46]. Although the precise mechanisms of shuttling of the ubiquitinated nascent chain to the proteasome are not yet understood, TOM1 has been implicated as it binds to the nascent chain, the proteasome, and the p97‐UFDL1‐NPLOC1 complex in the ribosome‐free state [47].

### Translational repression and degradation of the mRNA

In addition to promoting ribosomal splitting through 40S ubiquitination, ZNF598 also recruits the GIGYF2‐4EHP translational repressor complex to the mRNA. GIGYF2 and 4EHP induce localized cap‐dependent translation repression by sequestering the 5′ cap, thereby reducing the likelihood of further collisions on the same mRNA [48]. The mRNA may also be degraded through a pathway known as no‐go decay (NGD). NONU‐1 in *C. elegans* [49] and Cue2 in yeast are the key endonucleases involved in NGD which specifically targets the mRNA at collided ribosomes [49, 50]. The role of mammalian homolog to Cue2, N4BP2, in this mechanism, is not clear, although it associates with translating polyribosomes [51]. The fragments produced from these endonuclease cleavages are then rapidly degraded by Xrn1 and the exosome [50].

### Alternative/parallel RQC pathways

While the ZNF598‐mediated “canonical” RQC pathway is highly conserved in eukaryotes, it is not the sole possible cellular response to ribosome stalling. Indeed, several alternative sensors and pathways have evolved that mediate distinct responses to ribosome collisions. Initiation RQC (iRQC) is a variation of the RQC pathway that is triggered by ubiquitin ligase RNF10 [52]. RNF10 differs from ZNF598 in that it can target both stalled elongating 80S ribosomes and blocked initiating ribosomes (PIC) by monoubiquitinating the 40S subunit at eS2 and eS3 [52]. This ubiquitination appears to target 40S substrates at stalled ribosomes for degradation while the 60S is recycled [52].

The RNA‐binding and E3 ubiquitin ligase Makorin Ring Finger Protein 1 (MKRN1) binds to cytoplasmic poly(A)‐binding protein PABPC1, upstream of poly(A) sequences where it directly ubiquitinates PABPC1 and eS10, thus inducing stalling of the ribosome before it translates the poly‐A sequence [53]. This stalling leads to ZNF598‐mediated RQC and resolution of the collided ribosomes. Similar to ZNF598, endothelial differentiation‐related factor 1 (EDF1) also detects collided ribosomes, recruiting GIGYF2 and 4EHP to repress the cap‐dependent translation [48, 51, 54]. EDF1 also induces a transcriptional stress response programme through upregulation of transcription factors such as c‐JUN [51].

**Fig. 2:**
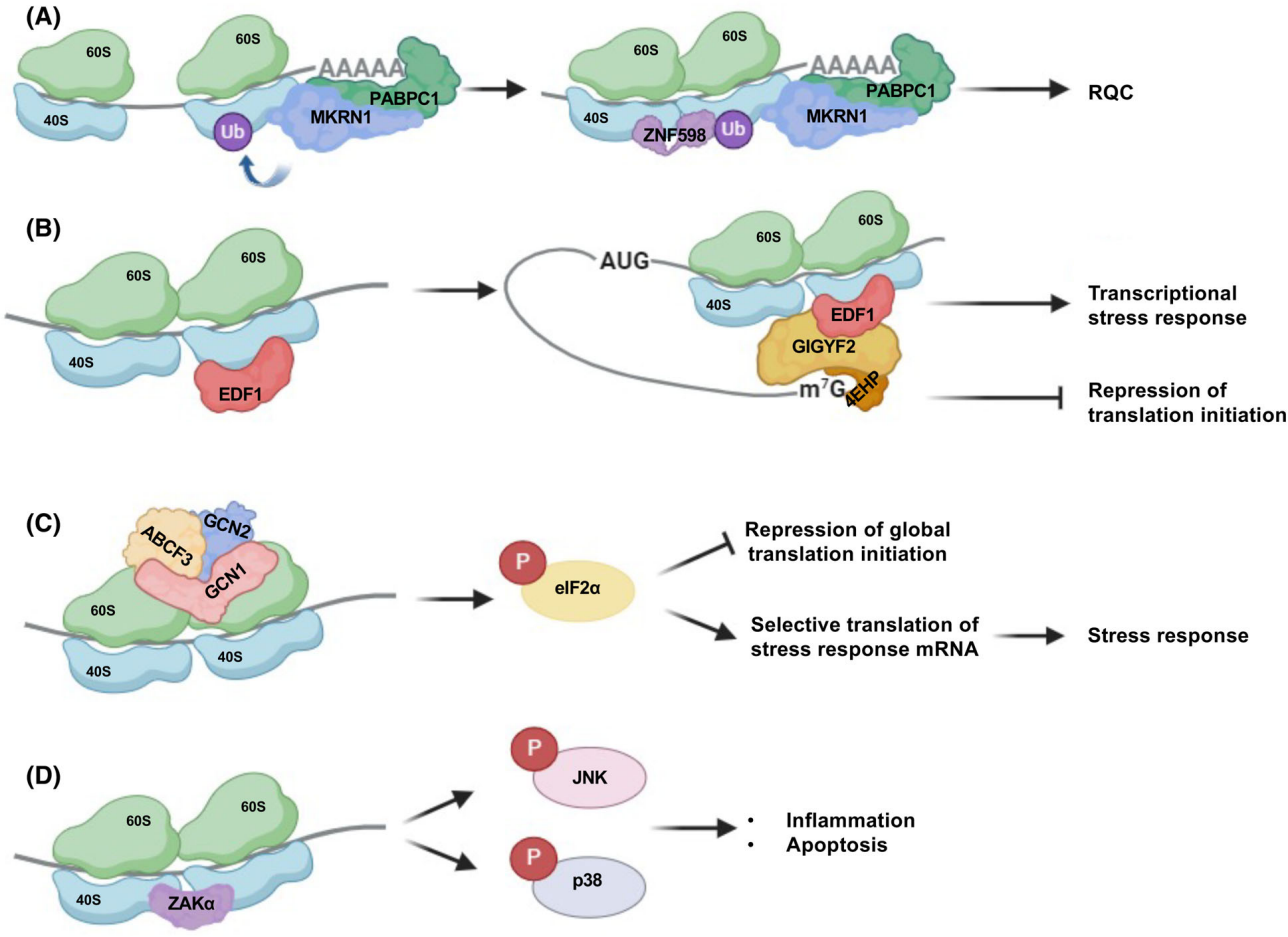
Alternative Ribosome Quality Control pathways. (A) MKRN1 binds to cytosolic poly‐A binding protein PABC1 at premature polyadenylation sites on mRNA and ubiquitinates the uS10 (RPS10) subunit of 40S, inducing ribosomal stalling before the polyadenylated sequence is translated. This leads to ribosomal collision and initiates “canonical” RQC via ZNF598, as described in Fig. 1. (B) EDF1 binds to collided ribosomes, inducing a c‐JUN mediated transcriptional response, and recruits the GIGYF2/4EHP complex which inhibits cap‐dependent translation of the faulty mRNA. (C) GCN2 is recruited to collide ribosomes along with GCN1 and ABCF3, the human homolog of GCN20 in yeast, initiating the Integrated Stress Response (ISR) through phosphorylation of eIF2α. Phosphorylated eIF2α induces a general shutdown of cap‐dependent translation initiation but increases the translation of mRNAs encoding proteins involved in stress response. (D) ZAKα binds to collided ribosomes, inducing the Ribotoxic Stress Response (RSR) by activating stress‐activated protein kinases (SAPKs) p38 and JNK. Activation of these SAPKs promotes inflammation and stress responses, including cell cycle arrest and eventually apoptosis after strong and sustained signalling.

The mitogen‐activated protein kinase ZAKα is an alternative sensor of ribosome collisions that can initiate either the integrated stress response (ISR) or the ribotoxic stress response (RSR) [55]. GCN2, one of the four known protein kinases that trigger ISR by phosphorylation of eIF2α is recruited to collided ribosomes with its coactivators, GCN1 and ABCF3 (the human homolog of GCN20 in yeast) through a mechanism that may involve ZAKα [55, 56]. GCN1 may also function as the preferred sensor for ribosome collisions occurring at the 3′ poly(A) tail upon stop codon readthrough [57, 58]. Following recruitment to collided ribosomes, GCN1/2 activates ISR by phosphorylation of eIF2α, leading to inhibition of general cap‐dependent translation [59]. Concurrently, ISR enables translation of specific mRNAs that encode stress response proteins [60, 61]. Alternatively, ZAKα can activate the stress‐activated protein kinases (SAPKs), JNK and p38, to induce RSR through a mechanism that may involve EDF1 [51, 55, 62]. RSR signalling leads to inflammation, cell cycle arrest, and eventually apoptosis after sustained signalling [55, 63]. Notably, RQC deficiency under collision‐causing conditions increases the SAPK signalling indicating that RSR can respond to the same type of collisions that RQC resolves [55]. It is still not fully understood what causes ZAKα to induce the RSR in certain circumstances and the ISR in others. It is plausible that lower levels of collisions could favour ISR activation that promotes a return to cellular homeostasis and cell survival while higher levels of collisions could lead to RSR that commits the cell to apoptosis (Fig. 2). Alternatively, these responses may be triggered sequentially depending on the kinetics and amplitudes of collided ribosomes [64].

## RQC and diseases; risks and opportunities

Ribosome stalling and collisions can occur in healthy organisms due to features such as truncated ORFs, lack of stop codons, or premature polyadenylation, which are estimated to impact a significant percentage of mRNAs [65]. The recognition that cells deploy a multipronged response to ribosome collisions, encompassing interventions at the site of collision, the translation of affected mRNAs, and broader impacts on general translation, underscores the significance of RQC in maintenance of cellular homeostasis. Thus, perhaps not surprisingly, disruptions in the RQC pathway and dysregulated expression or mutations in key “RQC factors” have been implicated in various human diseases (Table 1). Here, we will focus on the growing body of evidence implicating “RQC factors” in diseases. These encompass mutations or dysregulated expression of the components of the RQC pathways that are linked with the disease pathology, supported by observation of relevant phenotypes in genetically modified animal models.

**Table 1:** Summary of evidence that links dysregulated RQC to diseases.

Factor	Molecular function	Link to diseases
Neurological disorders		
LTN1	E3 ubiquitin ligase; targets nascent polypeptide chains to proteasome	Neurite outgrowth and morphogenesis are compromised in *Ltn1*‐depleted mouse primary neurons [71]
		*Ltn1* mutation leads to severe neurological abnormalities in mice [72]
NEMF	Binds to stalled 60S ribosomal subunit and promotes the recruitment of LTN1 for ubiquitination of nascent chains	*Nemf* knockdown leads to impairment of axonal outgrowth and synapse development in cultured mouse primary cortical neurons and biallelic *NEMF* variants result in central nervous system impairment in humans [73]
		NEMF is enriched in amyloid plaque nuclei in brain tissue from Alzheimer's disease patients [99]
		*NEMF* mutations are associated with neurodegeneration and neuromuscular disease in mice and humans [96]
ZNF598	E3 ubiquitin ligase; detects the collided ribosomes & mediates ubiquitination of small ribosomal subunits	ZNF598 supresses cellular pathologies in ALS patient‐derived neurons [91]
		ZNF598 is enriched in amyloid plaque nuclei in brain tissue from Alzheimer's disease patients [99]
ANKZF1	Endonuclease; cleaves polypeptidyl‐tRNAs to release polypeptides for degradation	ANKZF1 is enriched in amyloid plaque nuclei in brain tissue from Alzheimer's disease patients [99]
GTPBP1	GTPase; facilitates the release of peptidyl‐tRNA from the stalled ribosome	Loss of *Gtpbp1* leads to widespread neurodegeneration in mice [210]
GTPBP2	GTPase; involved in releasing the peptidyl‐tRNA from stalled ribosomes	*GTPBP2* mutation leads to neurodegeneration and intellectual disability [68], and Jaberi‐Elahi syndrome [69, 211] in humans
		Depletion of *Gtpbp2* leads to widespread neurodegeneration in mice [70]
p97/VCP	ATPase; removes ubiquitinated nascent chains from stalled ribosomes and directs them to proteasome	*VCP* mutations are identified in humans with neuromuscular diseases [97]
		*VCP* mutation is associated with Paget's disease of bone and FD in human [212]
ABCE1	ATPase; recycles stalled ribosomes	ABCE1 prevents mitochondrial protein aggregation and neuronal loss in mammalian & *Drosophila* PD models [87]
		ABCE1 expression is downregulated in PD patient's brain tissue [86]
GIGYF2	Inhibits the translation of defective mRNAs	Disruption of *Gigyf2* causes neurodegeneration in mouse spinal anterior horn motor neurons [74]
		*Gigyf2* mutations are detected in PD patients [83, 84, 85]
Developmental and congenital disorders		
LTN1	E3 ubiquitin ligase; targets nascent polypeptide chains to proteasome	*Ltn1*‐deficiency leads to dendritic and synaptic abnormalities along with cognitive disorders in mice [109]
GIGYF2	Inhibits the translation of defective mRNAs	*Gigyf2* is crucial for normal embryonic development. Its disruption causes early postnatal death in mice [74]
PELO	Splits the stalled ribosome	*Pelo*‐deletion affects prenatal cerebellar neurogenesis and leads to neurodevelopmental deficit in mice [107]
		*Pelo* knockdown disrupts epidermal homeostasis in mice [108]
HBS1L	GTPase; splits the stalled ribosome	*Hbs1l* depletion leads to neurodevelopmental pathology [105], prenatal cerebellar neurogenesis [107], and retinal dystrophy [205] in mice
ZAKα	MAPKKK; activated by ribosome collisions, initiates RSR pathway	*Zakα* mutants are associated with abnormal limb development in human [106]
Metabolic disorders		
ZAKα	MAPKKK; activated by ribosome collisions, initiates RSR pathway	ZAKα deficiency leads to decreased lifespan in *C. elegans* [62]
		*ZAKα* depletion relieves the ROS‐induced lethality in zebrafish larvae and protects against obesity‐associated metabolic dysfunction in mice [122]
Cancer		
p97/VCP	ATPase; removes ubiquitinated nascent chains from stalled ribosomes and directs them to proteasome	p97 expression is increased in breast cancer stem cells and correlates with poor prognosis [149]
ZNF598	E3 ubiquitin ligase; detects the collided ribosomes & mediates ubiquitination of small ribosomal subunits	ZNF598 depletion leads to increased 5FU‐induced cell death in human colorectal cancer and pancreatic cancer cells [151]
ASCC3	Helicase; involved in resolving stalled ribosomes and aiding in the degradation of problematic nascent polypeptides	ASCC3 is upregulated in non‐small cell lung cancer and correlates with poor pathological features and prognosis [153]
ANKZF1	Endonuclease; cleaves polypeptidyl‐tRNAs to release polypeptides for degradation	ANKZF1 expression is associated with prognosis in colorectal cancer [154]
		ANKZF1 knockdown leads to reduced malignant progression of glioblastoma cells [160]
ABCE1	ATPase; recycles stalled ribosomes	Suppression of ABCE1 significantly reduces the growth and invasiveness of neuroblastoma cells and patient‐derived xenograft tumours [155]
RACK1	Subunit of 40S ribosome, detects ribosome stalling, recruits other RQC factors upon stalling	RACK1 expression is associated with poor prognosis in breast [156] and lung cancers [157]
LTN1	E3 ubiquitin ligase; targets nascent polypeptide chains to proteasome	Overexpression of LTN1 in hepatocellular carcinoma cells suppresses cell proliferation *in vitro* and *in vivo* [158]
GIGYF2	Inhibits the translation of defective mRNAs	GIGYF2 expression is associated with glioma malignancy and better patient survival [159]
GCN2	Kinase; triggers the ISR pathway to modulate mRNA translation in response to ribosome collisions	GCN2/p‐eIF2α/ATF4 pathway is essential to cancer growth in response to nutrient deprivation [161, 162]
Response to viral infections		
ZNF598	E3 ubiquitin ligase; detects the collided ribosomes & mediates ubiquitination of small ribosomal subunits	*Znf598* deletion inhibits Vaccinia [199] and Poxvirus [188] virus replication
		ZNF598 regulates interferon‐stimulated gene expression [188]
RACK1	Subunit of 40S ribosome, detects ribosome stalling, and recruits other RQC factors upon stalling	RACK1 is required for postreplicative poxvirus protein synthesis [213]
GIGYF2	Inhibits the translation of defective mRNAs	GIGYF2 depletion decreases viral replication rate due to derepression of Interferon ß production [189]

### Neurodegeneration and neurological disorders

Dysregulated proteostasis and accumulation of protein aggregates are hallmarks of several neurological disorders [66, 67]. Neurons are particularly susceptible to protein misfolding and aggregations due to their long lifespan, limited regenerative capacity, intricate synaptic connections, and restricted immune clearance. Thus, a dysfunctional RQC may disproportionately impact the development and functions of neurons and thereby induce neurological disorders.

Several lines of evidence suggest a robust and causal link between RQC and neurodegeneration. For instance, disruptive mutations in *GTPBP2* gene have been identified in members of several unrelated families that exhibited neurodegenerative features [68, 69]. Depletion of GTPBP2, a PELO interacting protein, in a mouse model that carries a second mutation in a tRNA‐Arg that is expressed predominantly in the central nervous system, leads to accumulation of ribosomal stalling at arginine codons and widespread neurodegeneration [70]. Depletion of *Ltn1* or its RING domain, which abrogates the E3 ligase activity, significantly impairs the morphogenesis of mouse primary neurons *in vitro* due to aberrant degradation of the faulty nascent peptides [71]. Consistently, mice with homozygous deletion of the *Ltn1* gene exhibit profound early‐onset and progressive neurological and motor dysfunction [72]. Loss of *Nemf* also impairs axonal outgrowth and synapse development in cultured mouse primary cortical neurons [73] and biallelic *NEMF* mutations have been identified in families with hereditary neurological disorders [73].

Heterozygous deletion of *Gigyf2*(+/−) in mice results in neurodegeneration in spinal anterior horn motor neurons and presence of cytoplasmic Lewy body‐like inclusions [74]. Lewy bodies are composed of a dense filamentous and granular material and are observed in several neurological disorders such as Alzheimer's and Parkinson's diseases [75]. It should be noted that besides its role in RQC, GIGYF2 also impacts mRNAs targeted by other mechanism such as microRNA‐mediated silencing [76]. Thus, further studies are needed to assess which function(s) of GIGYF2 is critical for prevention of these neurotoxic phenotypes.

### Huntington's disease

Huntington's disease is a fatal neurodegenerative disorder caused by expansion of CAG trinucleotide repeats in the Huntingtin (*HTT*) gene, leading to the inclusion of a polyglutamine (polyQ) tract in the encoded protein. Presence of the CAG repeats in the *HTT* mRNA alters the elongation kinetics and causes ribosome collisions. Thus, the mutant HTT (mHTT) protein is prone to aggregation and a contributor to the neurotoxic phenotype [77]. Furthermore, translation of the *mHTT* mRNA exacerbates ribotoxicity through sequestration of the elongation factor eIF5A. Reduction in available eIF5A aggravates ribosome stalling on eIF5A‐dependent sites (typically Proline‐rich motifs) and impairs co‐translational proteostasis and stress responses [78]. In a yeast model, deletion of *Ltn1* and *Rqc1* (yeast homolog of TCF25) leads to nuclear accumulation of mHTT [79]. Ltn1 is also crucial in protecting yeast cells against the cytotoxic effects of mHTT, including altered actin cytoskeletal structures and abrogated endocytosis [72, 80]. Importantly, an increase in mRNA translation, due to hyperphosphorylation of the translation inhibitor eIF4E‐binding protein 1 (4E‐BP1), has been observed in Striatal projection neurons from HD patients, and treatment with the translation initiation inhibitor 4EGI‐1 prevented the motor deficits symptoms in mice [81].

### Parkinson's disease

Parkinson's disease (PD) is a progressive neurodegenerative disorder that primarily affects the motor system. It involves the loss of dopaminergic neurons and dopamine deficiency, impacting movement and coordination. Identification of mutations in *PINK1* in familial PD has led to the model that PINK1‐mediated mitophagy leads to the accumulation of faulty mitochondria and PD pathogenesis [82]. Notably, mutations in the *GIGYF2* gene, which maps to the PARK11 locus (chromosome 2q37), have also been linked to an increased risk for PD and identified in late‐stage PD and in PARK11 familial PD [83, 84, 85]. Triggering of RQC on nuclear‐encoded mRNAs responsible for generating mitochondrial outer membrane proteins during mitochondrial damage has been proposed as a signal for activating mitophagy by PINK1. In response to mitochondrial stress, ribosome stalling on these mRNAs leads to the ubiquitination of ABCE1 by NOT4. Ubiquitinated ABCE1 recruits autophagy receptors to the mitochondrial outer membrane, initiating the process of mitophagy. Differential expression analysis of brain tissues revealed a significant downregulation of both ABCE1 and HBS1L in samples derived from PD patients compared to healthy individuals [86]. This emphasizes the critical role of the RQC process in the pathogenesis of PD. Further studies showed that mitochondrial dysfunction in mammalian as well as *Drosophila* cells impairs translational termination and ribosome stalling on nuclear‐encoded mitochondrial mRNAs and generation of protein aggregates in the cytosol [87].

### Neuromuscular diseases

Amyotrophic lateral sclerosis (ALS) is a rapidly progressing neurological disease characterized by deterioration of motor functions. In most cases, ALS is linked to the expansion of the GGGGCC (G4C2) repeats in the Chromosome 9 open reading frame 72 (*C9ORF72*) gene. The number of repeats can vary from a few to thousands. Proteotoxicity stemming from the presence of arginine‐containing dipeptide repeats derived from mistranslated *C9ORF72* expansion transcripts represents a major source of neurotoxicity in ALS patients [88].

Part of the proteotoxicity of these arginine‐rich dipeptide‐containing proteins is due to their propensity to aggregation, which may be related to induction of ribosome stalling during the translation of these segments of the mRNA [89, 90]. Studies on neurons generated through re‐differentiations of induced pluripotent stem cells (iPSCs) derived from *C9ORF72* ALS patients revealed an impaired RQC activity in these cells, likely due to lower expression of ZNF598 [91]. Furthermore, due to lack of lysine residues, these nascent peptides are resistant to LTN1‐mediated ubiquitination and proteasomal degradation [92]. The translocase of the outer membrane (TOM) complex at the surface of mitochondria generally detects the positively charged amino acid sequences of mitochondrial targeting sequence (MTS) in the N terminus of nuclear‐encoded proteins and facilitates their importation into mitochondria [93]. The arginine‐rich sequence in *C9ORF72* expansion mimics the MTS and engages with the TOM complex, leading to importation of the nascent protein into mitochondria, and further toxicity [94]. Notably, translation of mRNAs that encode mitochondrial proteins near the outer mitochondrial membrane often results in ribosome stalling. Yet, the co‐translational importation of the nascent proteins renders the nascent peptides inaccessible for ubiquitylation and subsequent proteasomal degradation. To prevent the aggregation of these peptides, which otherwise could result in sequestering mitochondrial chaperones and toxicity, Vms1 (the yeast homolog of ANKZF1) binds to 60S ribosomes at the mitochondrial surface to prevent CAT‐tailing of proteins already engaged with import machinery. Instead, the nascent peptide will be imported into the mitochondria and degraded by the mitochondrial quality control [95].

In mice, mutations in *Ltn1* and *Nemf* genes lead to progressive motor neuron degeneration [72, 96]. Similarly, mutations in *P97/VCP* are linked to defects in the ubiquitylation/protein degradation pathway in motor neurons [97]. Consistently, mutations in *Nemf* as well as *P97/VCP* were identified in patients with neuromuscular diseases [96, 97]. Mice with heterozygote mutations for ZNF598‐interacting GIGYF2 protein also demonstrate motor dysfunction [74], although a specific role of defective RQC in this phenotype is not established.

### Alzheimer's disease

The prevalent model for Alzheimer's disease (AD) pathogenesis proposes that dysfunctional metabolism of the amyloid precursor protein (APP) leads to the formation of β‐amyloid (Aβ) plaques derived from Aβ_42_ cleavage products of APP that drive disease progression [98]. Inefficient RQC during *APP* mRNA translation produces CAT‐tailed species that accelerate the formation of Aβ plaques [99]. Ribosomes stall at the ER membrane during co‐translational translocation of APP C‐terminal fragment (APP.C99), leading to truncated species with CAT‐tails that are prone to aggregation. This causes endolysosomal/autophagy defects, seeding Aβ peptide aggregation. Overexpression of the rate‐limiting ribosome recycling factor ABCE1 or knockdown of ZNF598, which cause inefficient RQC and therefore resumption of translation by the stalled ribosome, can rescue the proteostasis failure, endolysosomal/autophagy dysfunction, and cognitive deficits in AD mouse models [99]. Brain tissue from AD patients also shows enrichment of ZNF598, NEMF, and ANKZF1 in the nuclei of amyloid plaques, consistent with the hypothesis that ribosome collision may contribute to the pathogenesis of AD [99].

Overall, these observations imply a critical role for RQC in alleviating the potential toxicity of aggregated proteins due to translation of abnormal mRNAs in neural cells. In line with this, the overexpression of SARS‐CoV‐2‐encoded non‐structural protein 1 (NSP1), which binds to the 40S ribosomal subunits and inhibits general mRNA translation [100], significantly alleviates neuromuscular degeneration caused by ribosome collision in models of Alzheimer's disease (AD), Parkinson's disease (PD), and amyotrophic lateral sclerosis (ALS) [101]. This is consistent with a role for general or transcript‐specific increased mRNA translation in aetiology of these disorders [9]. Further understanding of RQC's involvement in neurological disorders is of great importance as it could offer valuable insights into disease mechanisms and pave the way for novel therapeutic strategies.

### Developmental and congenital disorders

Human genetics and experimental evidence from animal models revealed that RQC deficiency is linked with developmental and neurodevelopmental deficits. Analysis of developing zebrafish embryos demonstrated temporal ZNF598‐regulated changes in RPS10 ubiquitination [102], which indicates various rates of ribosome collisions at different stages of embryonic development. Accumulation of stalled ribosomes has also been observed during the terminal differentiation of erythroid cells and platelets, due to downregulation of ABCE1 [103]. However, the exact relationships between these changes in ribosome collisions and developmental phases remain unknown. Further studies are required to deduce if these changes in ribosome stalling/collisions are by‐products or integral to the differentiation programme.

Rare biallelic loss of function mutations in *HBS1L* have been linked with developmental defects including global developmental delay, severe intrauterine growth restriction, microcephaly, submucous cleft palate and retinal pigmentary deposits [104]. Similar phenotypes were also observed in a *Hbsl1‐*deficient mouse model [105]. In humans, familial mutations in the sterile alpha motif (SAM) motif of ZAKα are associated with hearing loss and abnormal limb development [106]. Notably, while the CRISPR/Cas‐mediated knockout of the two *Zak* isoforms (*ZAKα* and *ß*) is embryonically lethal in mice, genetically engineered mice wherein the SAM domain of ZAKα is deleted are viable but develop hindlimb defects [106]. Mechanistic studies suggest that these SAM domain mutants acquire a constitutively active conformation [62]. Yet, it is not clear why the deleterious phenotype is manifested in these tissues. The special (tissue‐specific) and temporal developmental defects due to deficiency of other “RQC factors” has also been observed. Whereas depletion of *Pelo* and *HBS1L* significantly affects prenatal cerebellar neurogenesis, it does not influence survival of these neurons after development [107]. Furthermore, while Lgr5‐expressing mouse intestinal stem cells remain intact upon conditional deletion of *Pelo*, the Lrig1‐expressing epidermal stem cells exhibit hyperproliferation and abnormal differentiation [108].

RQC deficiency can also lead to severe neurodevelopmental deficits. Neurons wherein *Ltn1* mRNA expression is depleted exhibit developmental defects due to the upregulation of the tetratricopeptide repeat domain 3 (TTC3) protein and UFMylation signalling proteins UFM1, UFL1, and CDK5RAP3 [109]. This leads to increased UFMylation (a ubiquitin‐like posttranslational modification) of ribosomal protein RPL26, which promotes the proteasome‐mediated degradation of arrested polypeptides. Furthermore, TTC3 inhibits translation initiation in a process that may be facilitated by ubiquitination of RPS2 and thereby reduces the accumulation of translationally arrested products. However, aberrantly accumulated TTC3 protein causes dendritic abnormalities and reduced surface‐localized GABA_A_ receptors during neuronal development [109]. Consistently, *Ltn1*‐deficient mice showed accumulation of TTC3, resulting in dendritic and synaptic abnormalities, along with behavioural deficits associated with cognitive disorders [109].

Fragile X syndrome (FXS) is the most common inherited form of intellectual disability, characterized by abnormal dendritic spines, childhood seizures, and autistic behaviours. Excessive mRNA translation, particularly of the highly abundant and efficiently translated mRNAs that encode ribosomal proteins is cited as a potential contributing factor to FXS pathobiology [110, 111]. Loss of function of the Fragile X mental retardation protein (FMRP), that is essential for synaptic plasticity and neuronal development, causes FXS. FMRP stalls ribosomes along the coding regions of mRNAs that are linked to neuronal development, synaptic function, and autism, while loss of FMRP relieves these stalled ribosomes [112, 113, 114]. Although the precise mechanism by which FMRP affects ribosome stalling and its relevance to the known RQC mechanism is not clear, FMRP protein is enriched in isolated nuclease‐resistant disomes, which are typically formed by collided ribosomes [114]. High‐throughput interactome analyses identified potential interactions between FMRP and “RQC factors” such as ZNF598 [115] and ZAKα [55], which further indicate cross‐talk between FMRP‐mediated stalling and the RQC pathway.

### Metabolic stress and diseases

Multiple lines of evidence underscore an intricate interplay between metabolic stress and RQC that is vital for maintaining cellular homeostasis and responding to stress. Metabolic imbalances can disrupt mRNA translation and cause ribosome stalling through various mechanisms. These include stalling due to limited availability of specific amino acids or their corresponding tRNAs [116, 117, 118]. Oxidative and alkylating stresses can induce ribosome stalling and triggering of RQC. Such stressors can directly impact both RNAs and components of the translation machinery. For instance, alterations in cellular redox levels induced by treatment with the lipid electrophile 4‐hydroxy‐2‐nonenal [119] and reactive oxygen species (ROS) [120, 121] results in oxidation of ribosomal proteins and translation factors, altering their activities. Although the potential effects of these modifications on ribosome stalling and RQC activation are not fully understood, excessive generation of ROS has been shown to impair translation factors and tRNAs and trigger ribosome stalling and collision [122]. Oxidative stress‐induced modifications of ribosomal proteins could also disrupt translation elongation or termination [120] and stall the translating ribosomes. Consistent with this notion, treatment with the proteasome inhibitor MG132 prior to oxidative stress induced by hydrogen peroxide exposure partially rescues newly synthesized proteins, likely by inhibiting the RQC‐directed proteasomal degradation of the nascent peptides [120].

A variety of exogenous and endogenous chemicals, such as ROS, nitric oxide, hypohalous acids, and alkylating agents can damage ribonucleotide bases [123]. These have the potential to affect the function of both mRNAs and noncoding RNAs (e.g. rRNAs) by creating lesions such as bulky adducts, interfering with base‐pairing interactions, and altering the chemical properties of rRNAs. These modifications can impede the decoding ability of the ribosome, leading to ribosome stalling. RQC can resolve collided ribosomes formed on adducts, such as 8‐oxoG in mRNAs, that result from treatment with oxidizing and alkylating agents or UV radiation [124, 125]. Aldehydes, such as acetaldehyde and formaldehyde, are highly reactive compounds that are produced during cellular metabolism or after alcohol consumption but are normally catabolized and neutralized by the aldehyde‐clearing aldehyde dehydrogenases enzymes (e.g. ALDH2). Inactivation of aldehyde‐clearing enzymes, which occurs in patients with aldehyde degradation deficiency syndrome (ADDS) or their reduced expression with ageing [126], can lead to accumulation of aldehydes in tissues, leading to formation of adducts within biomolecules. RNA‐protein crosslinks due to treatment with aldehydes can result in ribosome stalling [127]. The GCN1/GCN2‐ and ZAKα‐mediated RQC are crucial for resolving the collided ribosomes caused by aldehyde‐induced RNA‐protein crosslinks and in maintaining cellular fitness [127, 128]. Excessive ribosome collisions, which trigger the GCN1/GCN2 mediated activation of ISR or ZAKα‐mediated activation of RSR can result in significant impacts on metabolic fitness. For instance, excessive production of nitric oxide leads to impairment of translation factors, ribosome stalling/collisions and activation of the ZAKα‐mediated RSR [129]. Activation of ISR and RSR may also be linked to the pathological conditions associated with increased ROS production. Accordingly, ribosome stalling and GCN2 activation due to excessive mitochondrial ROS production are involved in steroidogenic inhibition in mouse testes due to environmental stress [130]. ROS‐induced ribosome collisions on highly translated mRNAs were also shown to cause cytotoxic effects and lethality in developing zebrafish larvae. However, depletion of ZAK relieved ROS‐induced lethality in zebrafish larvae and protected mice against obesity‐associated metabolic dysfunction, including blood glucose intolerance and liver steatosis induced by a high‐fat high‐sugar diet [122].

There is ample evidence to link deleterious oxidative stress, coupled with a decline in cellular proteostasis to the ageing process [131]. Ageing alters the kinetics of translation elongation and increases the likelihood of ribosome stalling at Arg, Lys and Pro residues, which contributes to proteostasis impairment and systemic decline [132]. However, the precise role of RQC in mediating or protecting against the ageing symptoms is not well understood. An increase in ROS production during the ageing process is associated with ribosome stalling/collisions and activation of RSR [122]. However, ZAK knockout mice are protected against the hallmarks of metabolic ageing, such as impaired blood glucose intolerance, stochastic liver steatosis, and whitening of brown adipose tissue [122]. While absence of RSR can reduce certain cytotoxic phenotypes, it is not clear how organisms tolerate the potential cytotoxic impacts of unresolved stalled/collided ribosomes. In contrast, a deficiency in the RSR has been reported to compromise the lifespan of *C. elegans* [62].

Interestingly, chemical modulation of RQC on specific mRNAs may hold potential therapeutic value for certain metabolic disorders. PF846, initially identified as a specific inhibitor of the proprotein convertase subtilisin/kexin type 9 (PCSK9), directly binds to ribosomes and selectively induces stalling on *PCSK9* mRNA and a limited number of other mRNAs [133]. PCSK9 promotes the lysosomal degradation of the low‐density lipoprotein receptor (LDL‐R), which facilitates the uptake of low‐density lipoprotein‐cholesterol (LDL‐C) from plasma into hepatic cells. Elevated LDL‐C levels in plasma are a well‐established risk factor for cardiovascular diseases, and inhibition of PCSK9 production by PF846 reduces the total cholesterol and LDL‐C levels in preclinical models following oral dosing [134].

### Cancer

The accelerated cell proliferation in cancers requires substantial cellular resources, including increased protein synthesis and ribosome content. Thus, during oncogenic transformation, overall translation activity and ribosome biogenesis are often elevated [135], partly due to activation of signalling pathways such as mTORC1, which stimulate these processes (reviewed in ref. [8]). Considering the increased likelihood of ribosome collisions on highly translated mRNAs [122, 136, 137, 138], this increased rate of translation may also augment the risk of ribosome collisions in cancer cells.

Metabolic stresses due to oxidative agents such as ROS and nitric oxide are elevated in cancer cells [139], further increasing the risk of ribosome stalling caused by chemical modifications to mRNAs, ribosomes, and translation factors. Excessive production of aberrant mRNAs, such as abnormally spliced [140] and polyadenylated mRNAs [141], and dysregulated RNA modifications such as pseudouridination [142] in cancer cells can further exacerbate the potential for ribosome stalling. Furthermore, anti‐cancer treatments including radiotherapy [143] and chemotherapeutic agents such as oxaliplatin, gemcitabine, and 5‐Fluorouracil, are known to impact various aspects of RNA metabolism, including transcription, processing, and translation [144, 145, 146]. Interestingly, ribosome collisions at rare leucine‐encoding UUA codons and activation of ZAKα may play an important role in toxicity induced by DNA‐damaging anti‐cancer agents such as Etoposides. Accordingly, upon DNA damage, the transfer RNase SLFN11 cleaves and reduces the abundance of tRNA^UUA^, leading to ribosome stalling and triggering of the ZAKα‐mediated RSR and cell death [147]. However, while a causal role for general mRNA translation in tumourigenesis is well‐established [148], the consequences of ribosome stalling/collisions and RQC in tumourigenesis or cancer cells' response to treatments that alter RNA metabolism are ill‐defined.

Mutations or dysregulated expression of several “RQC factors” have been observed in various types of cancers. For instance, increased expression of p97/VCP is linked to tumourigenesis, and several inhibitors of p97/VCP have shown promising anti‐cancer effects [149]. However, p97/VCP also plays a role in other mechanisms besides RQC, such as the Endoplasmic Reticulum‐Associated Protein Degradation (ERAD) pathway, which complicates interpretation of its role in p97/VCP‐dependent RQC in cancer. Interestingly, activation of oncogenic HRAS or the AKT pathways, which lead to enhanced mRNA translation, results in increased proteotoxic stress and cell death upon p97/VCP inhibition [150]. This observation aligns with the model suggesting that cells with defective RQC mechanisms are more sensitive to treatments that elevate mRNA translation rates. Indeed, consistent with this model, *Znf598* knockout RQC‐deficient cancer cells display higher sensitivity to 5‐Fluorouracil‐induced cell death. Conversely, inhibition of mRNA translation with 4EGI‐I reduces the sensitivity of *Znf598* knockout cells to 5‐Fluorouracil [151]. Mutations and/or dysregulated expression of ASCC3 [152, 153], ABCE1 [155], RACK1 [156, 157], LTN1 [158], GIGYF2 [159], and ANKZF1 [154], have also been linked to various aspects of tumourigenesis. Subcutaneous or intracranial implanted tumour models showed that shRNA‐mediated downregulation of ANKZF1 results in reduced malignant progression of glioblastoma cells. This is due to the accumulation of CAT‐tails in mitochondria, leading to sequestration of the mitochondrial chaperones (e.g. HSP60 and mtHSP70) and respiratory chain subunits, thereby activating the mitochondrial unfolded protein response as well as causing dysfunctional mitochondrial oxidative phosphorylation and apoptotic cell death [160].

GCN2 is upregulated in a wide range of cancer types and the GCN2/p‐eIF2α/ATF4 pathway is essential to cancer growth in response to nutrient deprivation. Furthermore, GCN2 expression is required for nutrient scavenging‐dependent growth in pancreatic cells and an estimated ~ 13% of cancers are dependent on GCN2 [161, 162]. Similarly, RSR signalling has been implicated in cancer. ZAKα is highly upregulated in breast, colorectal, urinary, and gastric cancers and its expression is negatively correlated with survival [163, 164, 165, 166, 167, 168]. ZAKα's oncogenic activity is also associated with cell motility in colorectal cancer and with the epithelial‐mesenchymal transition (EMT) [165, 167]. However, given that both ISR and RSR pathways can be triggered via several types of stimuli besides ribosome collisions, additional studies are necessary to delineate the implications of their specific RQC‐related functions in cancer.

Pseudouridination is the most prevalent posttranscriptional modification in RNAs including mRNAs. Dysregulated mRNA pseudouridination, along with increased expression of methyltransferase enzymes (writers) such as PUS7 and DKC1 have been frequently observed in cancers. Consistently, inhibiting PUS7 or DKC1, which prevents RNA pseudouridine modification, has been observed to suppress tumourigenesis [169, 170]. Crucially, the presence of pseudouridines within the open‐reading frame can impede translation elongation and result in ribosome stalling [171, 172, 173]. Notably, the presence of another type of modified nucleoside, methylguanosine, which in addition to the 5′ cap may also occur in the open‐reading frame [174], can also affect translation elongation. Thus, it would be interesting to investigate the frequency and impact of ribosome stalling/collisions induced due to the presence of modified RNAs in cancer cells. Furthermore, deducing the role of RQC in the cellular response to environmental stress or treatments [175] that impact RNA modifications requires further investigation.

Ferroptosis is an iron‐dependent form of necrotic cell death. Cancer cells often prevent ferroptosis through upregulation of the selenoprotein glutathione peroxidase 4 (GPX4), which blocks ferroptosis by converting toxic lipid peroxides into nontoxic lipid alcohols [176]. Selenoprotein mRNAs typically contain an in‐frame stop codon that is reprogrammed to allow selenocysteine incorporation, instead of translation termination. However, under conditions of low selenocysteine, the ribosome may stall at the relevant codon. Limiting selenium in cancer cells has been shown to lead to the induction of ferroptosis through ribosome stalling and rapidly reduced expression of GPX4 [177]. Arsenic, being a toxic element, is considered a primary risk factor for many health problems, ranging from skin diseases to cancer [178]. Conversely, arsenic trioxide is a certified drug for several types of cancers such as acute promyelocytic leukaemia [179]. Interestingly, arsenic trioxide can bind to ZNF598 and displace the Zn^2+^ ions within its RING finger motif leading to inactivation of ZNF598 and the RQC mechanism [180]. These observations indicate potential opportunities for utilizing the RQC mechanism as a cancer cell vulnerability for development of anti‐cancer treatments.

### Innate immunity and response to viral infections

Viruses often rely on the host cell's mRNA translation machinery to facilitate infection and replicate within the host. Hence, they are sensitive to and interact with the cellular mechanisms of regulation of mRNA translation. Upon entering the host cell, viruses manipulate the cellular translation apparatus to favour the translation of viral mRNA over host mRNA by employing various strategies such as viral RNA modification, cap‐snatching, and modulation of host translation factors (reviewed in ref. [181]). The innate immune response serves as the body's first line of defence against invading pathogens, including viruses. It constitutes a rapid and nonspecific defence mechanism that is crucial for initiating the immune response and preventing the spread of infection. The main mechanisms involved in the innate immune response include the recognition of pathogen‐associated molecular patterns (PAMPs) by pattern recognition receptors (PRRs) [182]. This recognition triggers signalling pathways that lead to the production of pro‐inflammatory cytokines, chemokines, and Interferons, which coordinate the immune response and recruit immune cells to the site of infection. However, it is equally important to precisely regulate the innate immune response to avoid autoimmune disorders [183]. To maintain the delicate balance in the regulation of the innate immune response several intrinsic regulatory mechanisms have evolved, to prohibit the excessive production of pro‐inflammatory cytokines and “overshooting” of the immune system upon detection of an infection [184].

Recent evidence indicates that certain “RQC factors” may also participate in the regulation of innate immune response. Depletion of ASCC3, itself an interferon‐stimulated gene (ISG) [185], leads to increased expression of several other ISGs such as IFI44, RSAD2, and IFIT2 and reduced replication of several positive‐strand RNA viruses from the Flaviviridae, Togaviridae, and Picornaviridae families [186]. ASCC3 has also been identified as a host factor that facilitates SARS‐CoV‐2 infection [187]. Similarly, depletion of ZNF598 [188], GIGYF2 [189, 190] and its paralogue GIGYF1 [191], or their interacting protein 4EHP [189, 190, 192] leads to significantly higher levels of immune response to viral infections. This is likely due to repression of important cytokines such as Interferon ß, Interleukin 8 and Colony‐Stimulating Factor 2 (CSF2) [189, 193]. Interestingly, certain viral encoded proteins were shown to interact with the components of the ZNF598/GIGYF2/4EHP complex. For instance, the SARS‐CoV‐2 encoded Non‐Structural Protein 2 (NSP2) interacts with ZNF598, GIGYF2, and 4EHP as well as the components of the ASC‐1 complex (e.g. ASCC3) [194]. The interaction with NSP2 results in increased affinity of GIGYF2 for 4EHP, repression of *Ifnb1* mRNA translation and enhanced viral replication [189], as well as increased GIGYF2/4EHP mediated repression of microRNA target mRNAs [195]. Similar interactions between the Epstein–Barr virus‐encoded BGLF2 and ZNF598, GIGYF2, and 4EHP have also been observed [196]. However, the potential impacts of these interactions on the efficacy of the RQC mechanism remain to be understood. Besides, ZNF598, GIGYF2 and GIGYF1 also interact with other RBPs such as Tristetraprolin (TTP) and the microRNA‐Induced Silencing Complex (miRISC), through which they can modulate the expression of many pro‐inflammatory cytokines [191, 193]. Further studies are required to establish a role for the RQC‐related functions of these proteins in regulating the expression of pro‐inflammatory cytokines and viral infection.

Certain evidence also suggests a more direct role for RQC in facilitating the infection and replication of DNA viruses. RQC deficiency leads to relocation of cGAS from the nucleus to the cytosol, activation of the cGAS/STING pathway, and increased production of the Interferon‐stimulated genes (ISGs). Notably, cGAS exhibits a preference for binding to collided ribosomes upon heightened levels of ribosome collisions [197]. Poxviruses, a family of double‐stranded DNA viruses that includes Vaccinia virus, possess an unusual 5′ UTR featuring an uninterrupted poly(A) sequence [198]. The phosphorylation of RACK1 by the viral B1 kinase promotes the translation of viral mRNAs, along with reporter mRNAs containing a poly(A) sequence within their 5′ UTRs. Significantly, Vaccinia infection triggers the ubiquitination of small ribosomal proteins, and the presence of functional ZNF598 is crucial for efficient viral mRNA translation and infection [188, 199]. In contrast, other evidence suggests that RQC may also contribute to repression of viral infection and an augmented activation of the immune response upon detection of viral infection. Viperin is another ISG that also leads to broad‐spectrum repression of translation of cellular as well a viral mRNAs, thereby blocking viral replication. This is achieved by catalysing the conversion of cytidine triphosphate (CTP) to 3'deoxy‐3′,4′‐didehydro‐CTP (ddhCTP), which randomly incorporates into RNAs and triggers ribosome collision with activation of the ZAKα/GCN2‐mediated ISR and translational repression [200]. RQC may also block the Epstein–Barr virus (EBV) by preventing the translation of the EBV Nuclear Antigen 1 (EBNA1) mRNA in infected cells. To overcome this, the virally encoded ubiquitin deconjugase (vDUB) BPLF1 removes the ubiquitin residues on the 40S subunits and the nascent EBNA1 protein, which are catalyzed by ZNF598 and LTN1 respectively. This leads to the readthrough of the stalled ribosome on the viral mRNA and production of the full protein [201]. RQC also contributes to the activation of the CD8+ T cell‐mediated immune response to viral infection by facilitating the proteasomal degradation of the viral protein, which enhances their presentation on MHC class I [202].

## Concluding remarks

Multiple studies have identified mutations in “RQC factors” associated with a wide range of diseases. However, establishing a causal relationship between these mutations and potential defects in the RQC process within the relevant disease context is often lacking. In cases such as neurological disorders, this gap is sometimes bridged by using animal models where the factor of interest is deleted or mutated. Nonetheless, these models may not always faithfully replicate human disease, and deletion of the relevant gene does not necessarily recapitulate the phenotypes caused by specific mutations that are detected in patients. Additionally, in contexts such as cancer, studies with suitable mouse models are lacking, emphasizing the need for further investigation. Importantly, these studies are further complicated by subtle yet significant differences in the mechanism of RQC between humans and lower eukaryotes, which should be taken into account when extrapolating results gained in animal models.

An important consideration when attempting to define the role of RQC in maintaining homeostasis or contributing to diseases is to separate the contribution of RQC‐related functions of “RQC factors” in pathological conditions from the molecular functions of these proteins that are unrelated to RQC. For instance, ZNF598 is an E3 ubiquitin ligase, which besides targeting small ribosomal subunits can also impact ubiquitination of other proteins in ostensibly RQC‐unrelated mechanisms. By triggering the conjugation of the ubiquitin‐like protein FAT10 to critical residues in the cytoplasmic viral RNA sensor RIG‐I, ZNF598 effectively inhibits the polyubiquitination of RIG‐I on the same residues and thereby suppresses the downstream signalling for type I Interferon production [203]. Similarly, ZNF598 can stimulate polyubiquitination and proteasomal degradation of the critical transcription factor NRF2 and thereby affect macrophage‐associated inflammation and cartilaginous endplate degeneration [204]. However, there is no evidence that either function is related to the role of ZNF598 in RQC. This highlights the importance of verifying the RQC‐related function of these proteins when studying their role in biological and pathological processes.

Depletion or mutation of different “RQC factors” can have conspicuous special and temporal effects. For instance, HBS1l and PELO are essential for prenatal cerebellar neurogenesis in mice but are expendable for survival of these neurons after development [107]. Furthermore, conditional deletion of *Pelo* resulted in abnormal differentiation of epidermal stem cells but did not have a tangible effect on intestinal stem cells [108]. This may be a consequence of different gene expression profiles in various tissues. Yet, further empirical evidence is required to understand the mechanistic basis for these differences. Defining the differential dependencies of distinct tissues and cell types may significantly benefit future studies into the development of therapies that target dysregulated RQC in various diseases and the potential side effects of such interventions.

Our understanding of the mechanisms of regulation of the RQC pathway and expression of “RQC factors” is still in its early stages. Evidence suggests that the stability of certain “RQC factors” may be coordinated, as seen in the simultaneous downregulation of PELO and EDF1 upon HBS1L depletion [205]. Coordinated mTOR‐regulated expression of ZNF598, GIGYF2, and ASCC3 has also been observed [151]. Additionally, mitochondrial stress [206] and treatment with the anti‐cancer chemotherapeutic drug 5‐Fluoruracil [151] lead to post‐translational stabilization of ZNF598. Furthermore, UV irradiation, which induces ribosome stalling and collisions [125] transcriptionally upregulates ZNF598 expression [207]. It would be interesting to establish whether there are intrinsic cellular mechanisms in place that coordinate the expression of at least some “RQC factors” in response to environmental stimuli that increase the risk of ribosome stalling to maintain cellular homeostasis.

Several lines of evidence indicate that reducing mRNA translation rate attenuates the negative impacts of defective RQC. This has been observed in Huntington's disease [81, 208], in *Pelo* knockout RQC‐deficiency phenotypes in mouse epidermal stem cells [108], and in 5FU‐induced toxicity in Znf598 cancer cells [151]. This is further supported by the observation that the SARS CoV‐2–encoded NSP1 protein, which blocks general translation initiation through binding to the PIC complex [100], prevents the symptoms of AD, PD, and ALS, which are associated with accumulation of protein aggregates due to ribosome stalling, but not other neurodegenerative disorders that are not known to be associated with ribosome stalling [101]. This may provide a promising opportunity for intervention in diseases where defective RQC is a contributing factor. As discussed above, several studies have already demonstrated the potential benefits of targeting RQC or modulating the rate of ribosome collisions in the treatment of neurological disorders, metabolic diseases, and cancer. However, it is important to note that modulation of RQC could also complicate the outcomes of other treatment regimes. For example, treatment with readthrough reagents for diseases caused by premature termination codons, as found in Cystic fibrosis and Hurler disease, may induce ribosome collisions at the 3′ poly(A) tail through GCN1 activation [57]. This underscores the necessity for further investigations into the causes and consequences of activating or inhibiting different branches of the RQC mechanism in order to maximize the benefits of treatments that affect RNA metabolism.

Despite significant advances in deciphering the mechanism of RQC and developing methodologies for detecting ribosome stalling and collisions, several key questions and technical limitations hinder our understanding of the role of RQC in maintaining homeostasis and in diseases. For instance, there is no clear‐cut distinction between ribosome pause and ribosome stalling, and it remains unclear why some cases of ribosome stalling trigger RQC while others do not. Additionally, the circumstances that trigger specific responses to collided ribosomes (e.g. ZNF598‐dependent RQC versus ZAK‐dependent RSR) are not clearly defined. Available methods for transcriptome‐wide analysis of ribosome stalling and collisions, such as Disome‐Seq, are complex, time‐consuming, and resource‐intensive. Moreover, these assays are presently only feasible in bulk tissues and lack the resolution needed to determine cell‐specific stalling and collisions. Recent advances in single‐cell Ribo‐Seq methods [209] may lay the groundwork for future development of essential tools for specialized analysis of ribosome stalling and collisions *in vivo*.

## Conflict of interest

The authors declare no conflict of interest.

## Author contributions

TM, OO, and SMJ wrote the manuscript.
